# Cyclooxygenase-2 enhances α2β1 integrin expression and cell migration via EP1 dependent signaling pathway in human chondrosarcoma cells

**DOI:** 10.1186/1476-4598-9-43

**Published:** 2010-02-23

**Authors:** Ju-Fang Liu, Yi-Chin Fong, Chih-Shiang Chang, Chun-Yin Huang, Hsien-Te Chen, Wei-Hung Yang, Chin-Jung Hsu, Long-Bin Jeng, Chih-Yi Chen, Chih-Hsin Tang

**Affiliations:** 1 Graduate Institute of Pharmaceutical Chemistry, China Medical University, Taichung, Taiwan; 2 Department of Orthopaedics, China Medical University Hospital, Taichung, Taiwan; 3 School of Chinese Medicine, China Medical University, Taichung, Taiwan; 4 Department of Orthopaedic Surgery, China Medical University Beigang Hospital, Yun-Lin County, Taiwan; 5 Department of Pharmacology, China Medical University, Taichung, Taiwan; 6 Department of Surgery, China Medical University Hospital, Taichung, Taiwan; 7 Cancer Institute, China Medical University Hospital, Taichung, Taiwan; 8 Graduate Institute of Basic Medical Science, China Medical University, Taichung, Taiwan

## Abstract

**Background:**

Cyclooxygenase (COX)-2, the inducible isoform of prostaglandin (PG) synthase, has been implicated in tumor metastasis. Interaction of COX-2 with its specific EP receptors on the surface of cancer cells has been reported to induce cancer invasion. However, the effects of COX-2 on migration activity in human chondrosarcoma cells are mostly unknown. In this study, we examined whether COX-2 and EP interaction are involved in metastasis of human chondrosarcoma.

**Results:**

We found that over-expression of COX-2 or exogenous PGE_2 _increased the migration of human chondrosarcoma cells. We also found that human chondrosarcoma tissues and chondrosarcoma cell lines had significant expression of the COX-2 which was higher than that in normal cartilage. By using pharmacological inhibitors or activators or genetic inhibition by the EP receptors, we discovered that the EP1 receptor but not other PGE receptors is involved in PGE_2_-mediated cell migration and α2β1 integrin expression. Furthermore, we found that human chondrosarcoma tissues expressed a higher level of EP1 receptor than normal cartilage. PGE_2_-mediated migration and integrin up-regulation were attenuated by phospholipase C (PLC), protein kinase C (PKC) and c-Src inhibitor. Activation of the PLCβ, PKCα, c-Src and NF-κB signaling pathway after PGE_2 _treatment was demonstrated, and PGE_2_-induced expression of integrin and migration activity were inhibited by the specific inhibitor, siRNA and mutants of PLC, PKC, c-Src and NF-κB cascades.

**Conclusions:**

Our results indicated that PGE_2 _enhances the migration of chondrosarcoma cells by increasing α2β1 integrin expression through the EP1/PLC/PKCα/c-Src/NF-κB signal transduction pathway.

## Background

Chondrosarcoma is the second most common malignancy of bone and it has a poor response to chemotherapy or radiation treatment currently-used, making the management of chondrosarcomas a complicated challenge [1]. Clinically, surgical resection remains the primary mode of therapy for chondrosarcoma. In the absence of an effective adjuvant therapy, this mesenchymal malignancy has a poor prognosis and therefore, it is important to explore novel and adequate remedies [2]. Since chondrosarcoma is a type of highly malignant tumor with a potent capacity to invade locally and metastasize distantly [2], an approach that decreases its ability to invade and metastasize may facilitate the development of effective adjuvant therapy.

Cyclooxygenases (COXs) are the rate-limiting enzymes that catalyze the conversion of arachidonic acid to prostaglandins (PGs). Two COX isoforms with distinct tissue distributions and physiological functions have been identified [3]. COX-1 is constitutively expressed in many tissues and plays important roles in the control of homeostasis [4]. Conversely, COX-2 is an inducible enzyme and is activated by extracellular stimuli such as growth factors and pro-inflammatory cytokines [5]. Recent investigations indicated that over-expression of COX-2 is frequently found in many types of cancer, including colon, lung, breast, pancreas, head and neck cancers [6-9], and is usually associated with poor prognosis and short survival. Identification of four subtypes of the PGE receptor (EP1-EP4) has made it possible to analyze their effects on human cancer cells [10,11]. Studies have shown that EP1 is coupled to Ca^2+ ^mobilization; EP2 and EP4 activate adenylate cyclase, whereas EP3 inhibits adenylate cyclase [12-14]. Furthermore, these studies indicated that cancer cells express multiple subtypes of the PGE receptor and that each subtype might be linked to different actions of PGE_2_.

Tumor invasion and metastasis are the critical steps in determining the aggressive phenotype of human cancers. Mortality in cancer patients principally results from metastatic spread of cancer cells to distant organs [15]. Integrins are a family of transmembrane adhesion receptors comprising 19α and 8β subunits that interact noncovalently to form up to 24 different heterodimeric receptors [16]. The combination of different integrin subunits on the cell surface allows cells to recognize and respond to a variety of different extracellular matrix proteins including fibronectin, laminin, collagen and vitronectin [16]. Activation and elevated expression of integrin-coupled signaling effectors have been implicated in the induction of a wide variety of human cancers, including those of the breast, colon, prostate, and ovaries [17-19]. In addition, integrin has also been implicated in metastasis of lung, breast, bladder and colon cancers [20-22].

The contribution of COX-2 to tumorigenesis has been intensively studied. Previous studies have shown that COX-2 modulates cell migration and invasion in several types of cancer cells [23,24]. Interaction of COX-2 with its specific EP receptors on the surface of cancer cells has been reported to induce cancer invasion [25]. However, the effect of COX-2 and EP receptors on migration activity in human chondrosarcoma cells is mostly unknown. Here we found the mRNA expressions of COX-2 and EP1 receptor in chondrosarcoma patients and chondrosarcoma cell lines were significantly higher than in normal cartilage. COX-2 and PGE_2 _also increased the migration and α2β1 integrin up-regulation of human chondrosarcoma cells. In addition, EP1 receptor, phospholipase Cβ3 (PLCβ3), protein kinase Cα (PKCα), c-Src and NF-κB signaling pathways were involved.

## Results

### COX-2 directed migration of chondrosarcoma cells via the EP1 receptor

**Figure 1:**
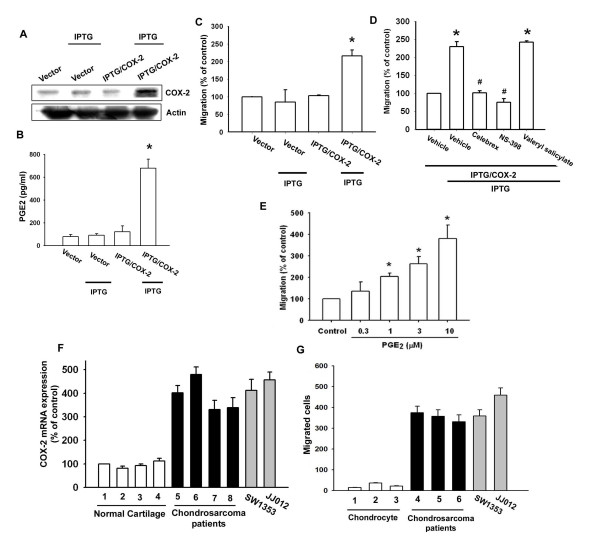
**COX-2-directed migration of human chondrosarcoma cells**. JJ012 cells were transfected with IPTG/COX-2 expression plasmid or control vector for 24 hr followed by stimulation with IPTG (5 mM) for 24 hr, the COX-2 expression, PGE_2 _production and migration activity were determined by Western blot analysis (A), ELISA assay (B) and Transwell (C). JJ012 cells were transfected with IPTG/COX-2 expression plasmid or control vector for 24 hr, and pretreated with valeryl salicylate (20 μM), celebrex (10 μM) or NS-398 (20 μM) for 30 min followed by stimulation with IPTG (5 mM), and *in vitro *migration was measured with the Transwell after 24 hr (D). JJ012 cells were incubated with various concentrations of PGE_2_, and *in vitro *migration activity measured with the Transwell after 24 hr (E). Total RNA were extracted from normal cartilage (lines 1-4), chondrosarcoma patients (lines 5-8) or from chondrosarcoma cell lines (SW1353 and JJ012), and subjected to qPCR analysis for COX-2 (F). The migration activity of each cells measured *in vitro *with the Transwell chamber after 24 h showed a significantly higher migration activity in primary chondrosarcoma and chondrosarcoma cell lines as compared with primary chondrocyte (G). Results are expressed as the mean ± S.E. *, p < 0.05 compared with control; #, p < 0.05 compared with PGE_2_-treated group.

COX-2 expression has been reported to stimulate directional migration and invasion of human cancer cells [23,24]. We used the IPTG-inducible COX-2 gene expression vector to examine the role of COX-2 in chondrosarcoma cells. JJ012 cells were transfected with IPTG-inducible COX-2 gene expression vector or control vector, and then IPTG (5 mM) was added for 24 hr. By Western blot analysis and ELISA, respectively, we found that IPTG induced COX-2 and PGE_2 _expression (Fig. 1A&1B). Furthermore, over-expression of COX-2 enhanced cell migration in chondrsarcoma cells (Fig. 1C). To confirm IPTG-inducible COX-2-mediated cell migration, the COX-2 specific inhibitors (celebrex and NS-398) were used. Celebrex and NS-398 but not COX-1 specific inhibitor (valeryl salicylate) reduced IPTG-inducible COX-2-mediated cell migration (Fig. 1D). We then directly exposed JJ012 cells to PGE_2 _and examined the migration activity. Stimulation of cells with PGE_2 _increased the migration activity in chondrosarcoma cells dose-dependently (Fig. 1E). We also examined human chondrosarcoma tissues for the expression of the COX-2 using qPCR. Expression of mRNA levels of COX-2 in human chondrosarcoma tissues (Fig. 1F, lines 5-8) and chondrosarcoma cell lines (SW1353 and JJ012) were significantly higher than those in normal cartilage (Fig. 1F, lines 1-4). Compared with normal cartilage, human chondrosarcoma tissues expressed a higher level of COX-2 mRNA (Table 1). In addition, primary chondrosarcoma cells and SW1353 or JJ012 cell lines were more migratory than normal chondrocyte (Fig. 1G). Thus, expression of COX-2 was associated with a metastatic phenotype of chondrosarcoma cells.

**Table 1:** Correlation levels of cyclooxygenase-2 (COX-2), EP1, α2 and β1 integrin expression in human chondrosarcoma and normal cartilage

	Normal cartilage				Chondrosarcoma						
**Patient**	**COX-2**	**EP1**	**α2 integrin**	**β1 integrin**	**Patient**	**Grade**	**Location**	**COX-2**	**EP1**	**α2 integrin**	**β1 integrin**

1	100%	100%	100%	100%	13	II	Pelvis	402%	378%	501%	399%
2	81%	127%	95%	99%	14	II	Scapular	479%	391%	389%	420%
3	93%	139%	112%	103%	15	II	Pelvis	331%	297%	391%	475%
4	112%	81%	98%	75%	16	II	Humerus	339%	289%	402%	512%
5	71%	66%	74%	86%	17	II	Humerus	597%	661%	505%	612%
6	55%	69%	67%	97%	18	II	Scapular	697%	788%	667%	669%
7	61%	55%	85%	92%	19	II	Pelvis	711%	791%	719%	739%
8	39%	41%	75%	66%	20	II	Pelvis	456%	505%	522%	712%
9	102%	112%	101%	108%	21	II	Scapular	421%	571%	565%	705%
10	111%	121%	112%	122%	22	II	Pelvis	397%	339%	631%	669%
11	91%	81%	77%	72%	23	II	Scapular	338%	397%	668%	612%
12	121%	108%	102%	105%	24	II	Pelvis	897%	721%	612%	579%
					25	I	Pelvis	322%	259%	339%	312%
					26	I	Pelvis	217%	267%	337%	332%
					27	I	Humerus	205%	201%	269%	279%
					28	I	Scapular	272%	288%	307%	309%
					29	I	Scapular	301%	322%	365%	331%

Results are expressed as % of patient 1

**Figure 2:**
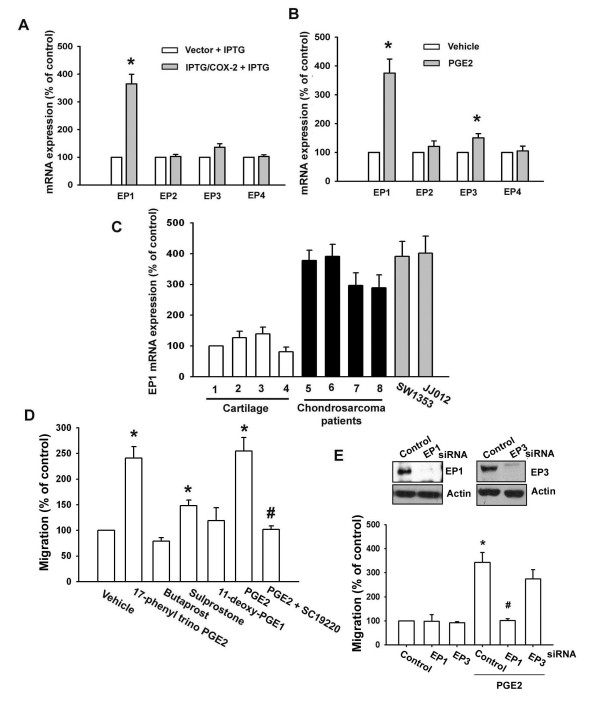
**EP1 receptor is involved in PGE_2_-mediated migration of human chondrosarcoma cells**. (A) JJ012 cells were transfected with IPTG/COX-2 expression plasmid or control vector for 24 hr followed by stimulation with IPTG (5 mM) for 24 hr, the mRNA expression of EP receptors was determined by qPCR. (B) JJ012 cells were incubated with PGE_2 _for 24 hr, and the mRNA expression of EP receptors was determined by qPCR. (C) Total RNA were extracted from normal cartilage (lines 1-4), chondrosarcoma patients (lines 5-8) or from chondrosarcoma cell lines (SW1353 and JJ012), and subjected to qPCR analysis for EP1 receptor. (D) JJ012 cells were 17-phenyl trinor PGE_2 _(3 μM), butaprost (10 μM), sulprostone (10 μM), 11-deoxy-PGE_1 _(10 μM) and PGE2 plus SC19220 (10 μM), and *in vitro *migration activity measured with the Transwell after 24 hr. (E) Cells were transfected with EP receptors siRNA for 24 hr followed by stimulation with PGE_2_, and *in vitro *migration measured with the Transwell after 24 hr. Results are expressed as the mean ± S.E. *, p < 0.05 compared with control; #, p < 0.05 compared with PGE_2_-treated group.

PGs exert their effects through interaction with specific EP1-4 subtype receptors [10-14]. To investigate the role of EP1-4 subtype receptors in COX-2-mediated increase of cell migration, we assessed the distribution of these EP subtype receptors in human chondrosarcoma cells by qPCR analysis. The mRNAs of EP1, EP2, EP3, and EP4 subtype receptors could be detected in human chondrosarcoma cells (Fig. 2A). After IPTG/COX-2-transfected JJ012 cells were treated for 24 hr with IPTG, the mRNA level of EP1 subtype receptor was increased, whereas EP2 and EP4 receptor mRNA remained un-changed (Fig. 2A). In addition, a similar induction of EP1 receptor mRNA, but not EP2 and EP4 receptor subtypes, was observed in JJ012 cells treated with PGE_2 _(Fig. 2B). However, over-expression of COX-2 and exogenous PGE_2 _slightly increased expression of EP3 receptor (Fig. 2A&2B). On the other hand, the mRNA levels of EP1 receptor in human chondrosarcoma tissues (Fig. 2C, lines 5-8) and chondrosarcoma cell lines (SW1353 and JJ012) were significantly higher than those in normal cartilage (Fig. 2C, lines 1-4). Compared with normal cartilage, human chondrosarcoma tissues expressed a higher level of EP1 mRNA (Table 1). To determine the role of EP1 receptor-dependent signaling in the regulation of cell migration in chondrosarcoma cells, the cells were treated with EP1-4-specific agonists, and then the cell migration activity was examined. Of the agonists tested, only the EP1/EP3-selective receptor agonist, 17-phenyl trinor PGE_2 _(3 μM), significantly increased the migration activity (Fig. 2D). In contrast, butaprost (EP2 agonist; 10 μM) and 11-deoxy-PGE_1 _(EP2/EP4-selective agonist; 10 μM) failed to up-regulate cell migration. Sulprostone (EP3 agonist; 10 μM) slightly increased cell migration in JJ012 cells (Fig. 2D). In addition, treatment with EP1 receptor antagonist SC19220 (10 μM) effectively antagonized the potentiating effect of PGE_2 _on cell migration activity (Fig. 2D). To further confirm this stimulation-specific mediation by EP1 receptor without EP3 receptor contamination, we assessed the role of EP1 and EP3 by using ON-TARGET smart pool EP1 and EP3, which decreases nonspecific effects by chemical modification and pooling [26]. Transfection of cells with ON-TARGET smart pool EP1 and EP3 siRNA reduced EP1 and EP3 expression, respectively (Fig. 2E; upper panel). Transfection of cells with EP1 but not EP3 siRNA effectively inhibited the PGE_2_-mediated migration of chondrosarcoma cells (Fig. 2E; lower panel). These results indicate that PGE_2 _increased cell migration in human chondrosarcoma cells via EP1 receptor.

### PGE_2_-directed migration of chondrosarcoma cells involves α2β1 integrin up-regulation

**Figure 3:**
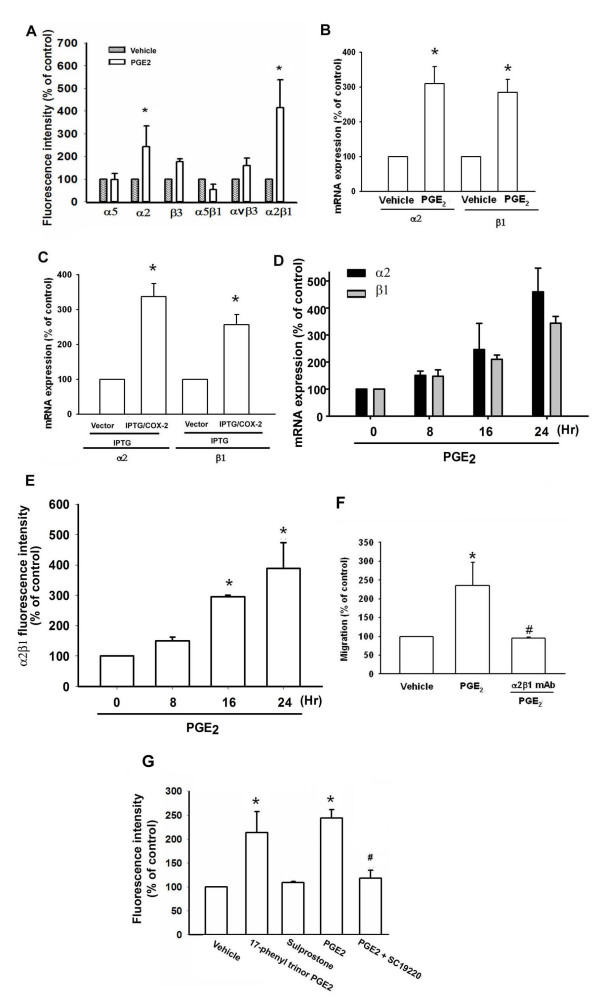
**COX-2-directed migration of human chondrosarcoma cells involves up-regulation of α2β1 integrin**. (A) JJ012 cells were incubated with PGE_2 _for 24 hr, and the cells surface α5, α2, β3, α5β1, αvβ3 and α2β1 integrin was determined using flow cytometry. (B) Cells were incubated with PGE_2 _for 24 hr, and the mRNA levels of α2 and β1 integrin was determined using qPCR. (C) JJ012 cells were transfected with IPTG/COX-2 expression plasmid or control vector for 24 hr followed by stimulation with IPTG (5 mM) for 24 hr, the mRNA expression of α2 and β1 integrin was determined by qPCR. JJ012 cells were incubated with PGE_2 _for indicated time intervals, and mRNA and cell surface α2β1 integrin were examined by qPCR (D) and flow cytometry (E). (F) Cells were pretreated with α2β1 monoclonal antibody (3 μg/ml) for 30 min followed by stimulation with PGE_2_. The *in vitro *migration activity measured after 24 hr. (E) JJ012 cells were treated with 17-phenyl trinor PGE_2 _(3 μM), 11-deoxy-PGE_1 _(10 μM), PGE_2_, and PGE_2 _plus SC19220 (10 μM), and cells surface α2β1 integrin was determined using flow cytometry. Results are expressed as the mean ± S.E. *, p < 0.05 compared with control; #, p < 0.05 compared with PGE_2_-treated group.

Previous studies have demonstrated significant expression of integrins in human chondrosarcoma cells [27]. We therefore, hypothesized that integrins may be involved in PGE_2_-directed migration of chondrosarcoma cells. Flow cytometry analysis showed that PGE_2 _induced the cell surface expression of α2 and α2β1 integrin in JJ012 cells (Fig. 3A). To confirm this finding, expression of mRNAs in the integrins in response to PGE_2 _was analyzed by qPCR. Treatment of JJ012 cells with PGE_2 _induced the mRNA expression of α2 and β1 integrins (Fig. 3B). In addition, treatment of IPTG/COX-2-transfected cells with IPTG increased mRNA expression of α2 and β1 integrins (Fig. 3C). Furthermore, compared with normal cartilage, human chondrosarcoma tissues expressed higher levels of α2 and β1 integrin mRNA (Table 1). Therefore, the α2β1 integrin plays an important role in PGE_2_-induced migration of human chondrosarcoma cells. Stimulation of cells with PGE_2 _also increased mRNA expression and cell surface expression of α2 and β1 integrin time-dependently (Fig. 3D&3E). Pretreatment of cells for 30 min with anti-α2β1 monoclonal antibody (mAb) (3 μg/ml) markedly inhibited the PGE_2_-induced cell migration (Fig. 3F). On the other hand, EP1/3 agonist enhanced the cell surface expression of α2β1 integrin (Fig. 3G). Pretreatment of cells with SC19220 reduced PGE_2_-mediated α2β1 integrin expression (Fig. 3G). These data suggest that PGE_2_-induced cancer migration may occur via activation of the α2β1 integrin.

### The PLC, PKC and c-Src signaling pathway is involved in PGE_2_-mediated integrin upregulation and cell migration of chondrosarcoma cells

**Figure 4:**
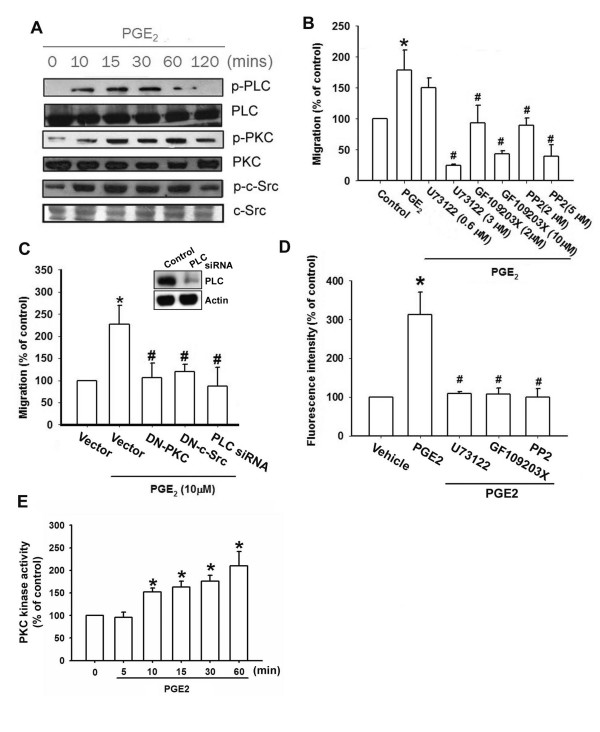
**PLC/PKC/c-Src signaling pathway is involved in PGE_2_-mediated migration and integrin upregulation in human chondrosarcoma cells**. (A) JJ012 cells were incubated with PGE_2 _for indicated time intervals, and p-PLCβ3, p-PKCα and p-c-Src expression was determined by Western blot analysis. (B&D) JJ012 cells were pretreated for 30 min with U73122, GF109203X or PP2. Then they were followed by stimulation with PGE_2_, and *in vitro *migration and cell surface α2β1 integrin were measured with the Transwell and flow cytometry after 24 hr. (C) Cells were transfected with PKCα or c-Src mutant and PLCβ siRNA for 24 hr followed by stimulation with PGE_2_, and *in vitro *migration measured with the Transwell after 24 hr. JJ012 cells were incubated with PGE_2 _for indicated time intervals, and PKCα activity was determined by the PKCα kinase assay kit. Results are expressed as the mean ± S.E. *, p < 0.05 compared with control; #, p < 0.05 compared with PGE_2_-treated group

It has been reported that PLC/PKC/c-Src dependent pathway is involved in EP1-mediated bone formation [28]. We therefore directly measured the phosphorylation of PLC, PKC and c-Src in response to PGE_2_. Treatment of JJ012 cells with PGE_2 _induced the phosphorylation of PLCβ3, PKCα and c-Src time-dependently (Fig. 4A). In addition, PKCα activity was also increased by PGE_2 _treatment of human chondrosarcoma cells time-dependently (Fig. 4E). Furthermore, pretreatment of cells with PI-PLC inhibitor (U73122), PKC inhibitor (GF109203X) and c-Src inhibitor (PP2) reduced PGE_2_-increased cell migration and integrin up-regulation (Fig. 4B&4D). Transfection of cells with PKCα and c-Src mutant or PLCβ siRNA also inhibited PGE_2_-mediated migration activity (Fig. 4C). Transfection of cells with PLC siRNA reduced PLC expression (Fig. 4C; upper panel). Based on these results, it appears that PGE_2 _acts through the PLCβ, PKCα and c-Src dependent signaling pathway to enhance α2β1 integrin expression and cell migration in human chondrosarcoma cells.

### NF-κB is involved in PGE_2_-mediated integrin upregulation and migration activity

**Figure 5:**
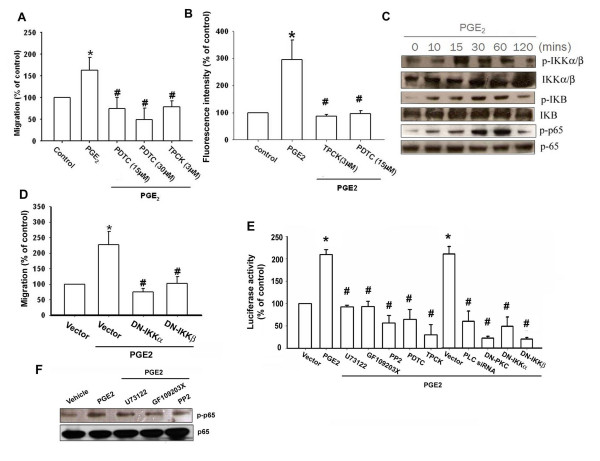
**PGE_2 _induces α2β1 integrin upregulation and cell migration through NF-κB activation**. Cells were pretreated with PDTC or TPCK for 30 min, then they were followed by stimulation with PGE_2_, and *in vitro *migration (A) and α2β1 integrin expression (B) were measured with the Transwell and flow cytometry after 24 hr. (C) JJ012 cells were incubated with PGE_2 _for indicated time intervals, p-IKK, p-IκBα and p-p65 expression was determined by Western blot analysis. (D) Cells were transfected with IKKα or IKKβ mutant for 24 hr followed by stimulation with PGE_2_, and *in vitro *migration measured with the Transwell after 24 hr. (E) Cells were pretreated with U73122, GF109203X, PP2, PDTC, TPCK, PDTC for 30 min or co-transfected with PLCβ siRNA, PKCα mutant, IKKα mutant, IKKβ mutant for 24 hr before incubation with PGE_2 _for 24 hr. The NF-κB activity was measured. (F) Cells were pretreated with U73122, GF109203X or PP2 for 30 min followed stimulation with PGE_2 _for 60 min, and p-p65 expression was examined by Western blot analysis. Results are expressed as the mean ± S.E. *, p < 0.05 compared with control; #, p < 0.05 compared with PGE_2_-treated group

As previously mentioned, NF-κB activation is necessary for the migration and invasion of human chondrosarcoma cells [27]. To examine whether NF-κB activation is involved in PGE_2_-induced cancer migration, an NF-κB inhibitor, PDTC, was used. Fig. 5A&5B show that chondrosarcoma cells pretreated with PDTC inhibited the PGE_2_-induced migration and integrin expression of chondrosarcoma cells. Furthermore, cells pretreated with TPCK (3 μM), an IκB protease inhibitor, also reduced PGE_2_-induced migration of cancer cells (Fig. 5A&5B). Therefore, the NF-κB pathway has a role in PGE_2 _induced migration of chondrosarcoma cells. We further examined the upstream molecules involved in PGE_2_-induced NF-κB activation. Stimulation of cells with PGE_2 _induced IKKα/β phosphorylation in a time-dependent manner (Fig. 5C). Furthermore, transfection with IKKα or IKKβ mutant markedly inhibited the PGE_2_-induced cell migration (Fig. 5D). These data suggest that IKKα/β activation is involved in PGE_2_-induced the migration activity of human chondrosarcoma cells. Treatment of chondrosarcoma cells with PGE_2 _also caused IκBα phosphorylation in a time-dependent manner (Fig. 5C). Previous studies showed that p65 Ser^536 ^phosphorylation increases NF-κB transactivation [29]. Therefore, the antibody specific against phosphorylated p65 Ser^536 ^was employed to examine p65 phosphorylation. Treatment of cells with PGE_2 _for various time intervals resulted in p65 Ser^536 ^phosphorylation (Fig. 5C). To directly determine NF-κB activation after PGE_2 _treatment, chondrosarcoma cells were transiently transfected with κB-luciferase as an indicator of NF-κB activation. As shown in Fig 5E, PGE_2 _treatment of chondrosarcoma cells for 24 hr caused increase in κB-luciferase activity. In addition, U73122, GF109203X, PP2, PDTC and TPCK or PLCβ siRNA, PKCα, IKKα and IKKβ mutant reduced PGE_2_-mediated NF-κB activity (Fig. 5E). Furthermore, U73122, GF109203X and PP2 reduced PGE_2_-mediated p65 phosphorylation (Fig. 5F). Taken together, these data suggest that activation of EP1 receptor, PLC, PKC and c-Src pathway is required for PGE_2_-induced NF-κB activation in chondrosarcoma cells.

## Discussion

The elucidation of the molecular biology of cancer cells in recent years has identified various molecular pathways that are altered in different cancers. This information is currently being exploited to develop potential therapeutic targets. To achieve metastasis, cancer cells must evade or co-opt multiple rules and barriers. Several discrete steps are discernible in the biological cascade of metastasis: loss of cellular adhesion, increased motility and invasiveness, entry and survival in circulation, exit into new tissue, and eventual colonization of a distant site [30]. The mechanism of metastasis is a complicated and multistage process. Here, we provide evidence that α2β1 integrin acts as a crucial transducer of cell signaling, regulating cell migration and COX-2 act as a critical mediator of the metastatic activity of cancer cells in the tumor microenvironment. In addition, α2β1 integrin mAb, U73122, GF109203X, PP2, PDTC, TPCK, PLC siRNA, PKC mutant, c-Src mutant, IKKα mutant and IKKβ mutant reduced PGE_2_-mediated cell migration in SW1353 cells (Additional file 1 - Figure S1). Furthermore, U73122, GF109203X, PP2, PDTC and TPCK also abolished PGE_2_-increased α2β1 integrin expression in SW1353 cells (Additional file 2 - Figure S2). Therefore, the same signaling pathways are involved in all chondrosarcoma cells. Moreover, α2β1 integrin mAb, U73122, GF109203X, PP2, PDTC, TPCK and EP1 siRNA reduced PGE_2_-mediated cell invasion in JJ012 cells [Transwell filters were precoated with Matrigel basement membrane matrix (BD Biosciences, Bedford, MA)] (Additional file 3 - Figure S3). Therefore, the same signaling pathways are involved in PGE_2_-mediated cell invasion in human chondrosarcoma cells.

COX-2 is a pleiotropic enzyme that mediates many physiological functions such as inhibition of cell apoptosis, augmentation of angiogenesis, as well as increased cell motility. These COX-2-mediated functions are mediated in part by various genes such as B-cell lymphoma-2 [31], myeloid cell leukemia-1, VEGF-A [32] and metalloproteinases [33]. However, the effect of COX-2 on migration activity in human chondrosarcoma cells is mostly unknown. Using qPCR analysis, we found that the expression of mRNA levels of COX-2 in human chondrosarcoma tissues and chondrosarcoma cell lines were significantly higher than those in normal cartilage. In this study, we used osteoarthritic cartilage to referee normal cartilage. However, cartilage from osteoarthritic patients may up-regulation COX-2 compared with normal cartilage. Therefore, the expression of COX-2 between normal cartilage, osteoarthritic cartilage and chondrosarcoma needs further examination. On the other hand, most of patient samples were isolated from low grade chondrosarcoma patients. Notably, grade 1 chondrosarcomas are not considered clinically overtly malignant or even locally aggressive lesion. Therefore, it might be possible that increased COX-2 expression was a result of inflammation for metaplasia. The expression of COX-2 in high grade chondrosarcomas are needed further examination. Moreover, primary chondrosarcoma cells and SW1353 or JJ012 cell lines were more migratory than normal chondrocyte. Our data provided the evidence that the expression of COX-2 is associated with a metastatic phenotype of chondrosarcoma cells. COX-2 exert it effects through interaction with specific EP1-4 receptors [10-14]. However, the expression of EP receptors in chondrosarcoma cells is largely unknown. We found that the chondrosarcoma cells expressed EP1-4 receptors. However, EP1 but not other EP receptors was required for PGE_2_-induced migration activity. Treatment with butaprost (EP2 agonist), and 11-deoxy-PGE1 (EP2/EP4 selective agonist) failed to up-regulate cell migration. Furthermore, EP1 but not EP3 siRNA inhibited PGE_2_-induced cell migration. Therefore, our data suggest a critical role for EP1 receptor in the PGE_2_-mediated cell migration in human chondrosarcoma cells.

Integrins link the extracellular matrix to intracellular cytoskeletal structures and signaling molecules and are implicated in the regulation of a number of cellular processes, including adhesion, signaling, motility, survival, gene expression, growth and differentiation [3,34]. Using flow cytometry analysis, we found that PGE_2 _increased α2β1 but not α5, β3, α5β1 or αvβ3 integrin expression, which plays an important role during tumor metastasis. Furthermore, PGE_2 _also increased the mRNA levels of α2 and β1 integrins. In addition, over-expression COX-2 increased the mRNA expression of α2 and β1 integrins. It has been often reported that α2β1 integrin has revealed the ability to act as critical molecules as regards metastasis the ability of chondrosarcoma cells [35]. In addition, activation of α2β1 integrin intracellular signal increased migration activity of chondrosarcoma cells [35]. Similarly, it was found that elevated expression of Cyr61 induced gastric cancer cell migration through α2β1 integrin [36]. Kawashima et al., also reported that tumour necrosis factor alpha induced migration of osteosarcoma cells via α2β1 integrin [37]. Collectively, our data also reveal that COX-2 and its downstream effector integrin α2β1, could constitute a potential target for future treatment of metastasis of chondrosarcoma cells.

It has been reported that PLC/PKC/c-Src dependent pathway is involved in EP1 receptor signaling [28]. In present study, we found PGE_2 _increased PLCβ3 phosphorylation in JJ012 cells. Several isoforms of PKC have been characterized at the molecular level and these have been found to mediate several cellular molecular responses [38]. We demonstrated that PKC inhibitor GF109203X antagonized the PGE_2_-mediated potentiation of migration activity and integrin expression, suggesting that PKC activation is an obligatory event in PGE_2_-induced α2β1 integrin expression in these cells. This was further confirmed by the result that the dominant negative mutant of PKCα inhibited the enhancement of migration activity by PGE_2_. Src, a tyrosin kinase, plays a critical role in the induction of chemokine transcription [39]. In human aortic endothelial cells, oxidized phospholipids induce IL-8 expression through c-Src activation [39]. As c-Src has been reported to be a downstream effector of G protein-coupled receptor [40], we examined the potential role of c-Src in the signaling pathway of PGE_2_-induced cell migration and integrin expression. We found that treatment of chondrsarcoma cells with PGE_2 _induced increases in c-Src phosphorylation at Tyr416. Taken together, our results provide evidence that PGE_2 _up-regulates cell migration and integrin expression in human chondrosarcoma cells via the EP1/PLC/PKCα/c-Src signaling pathway.

## Conclusions

The prognosis for patients with chondrosarcoma distant metastasis is generally considered very poor; hence, prevention of human chondrosarcoma metastasis is very important. In our study we observed that COX-2 increases the activity of α2β1 integrin via the EP1, PLC, PKCα, c-Src, and NF-κB-dependent pathway and enhances migration of human chondrosarcoma cells. Furthermore, the discovery of COX-2-mediated signaling pathway increases our understanding of the mechanism of human chondrosarcoma metastasis and may help us to develop more effective therapies in the future. Taken together, our data suggest that COX2/EP1 interaction plays a novel role in regulating chondrosarcoma cell migration/invasion in a clinical/experimental setting, and it would also appear to be feasible as a biological marker to predict the relative likelihood/extent of metastasis following chondrosarcoma cell migration.

## Methods

### Materials

Anti-mouse and anti-rabbit IgG-conjugated horseradish peroxidase, rabbit polyclonal antibodies specific for PLCβ, PKCα, c-Src, IKK, p-IκBα, IκBα, p65 and the siRNAs against PLCβ and control (negative control for experiments using targeted siRNA transfection; each consisted of a scrambled sequence that would not lead to the specific degradation of any known cellular mRNA) were purchased from Santa Cruz Biotechnology (Santa Cruz, CA, USA). Rabbit polyclonal antibody specific for p-IKKα/β, p-PLCβ3, p-PKCα, p-c-Src and p-p65 were purchased from Cell Signaling and Neuroscience (Danvers, MA). Rabbit polyclonal antibodies specific for α2, α5, β3, αvβ3, α5β1 and α2β1 integrin were purchased from Chemicon (Temecula, CA). PGE_2_, 17-phenyl trinor PGE_2_, butaprost, sulprostone, 11-deoxy-PGE_1_, SC19220 and rabbit polyclonal antibody specific for COX-2, EP1 and EP3 were purchased from Cayman Chemical (Ann Arbor, MI). Valeryl salicylate, NS398, U73122, GF109203X, PP2, PDTC, TPCK and IPTG (isopropyl-β-D-thiogalactopyranoside) were obtained from Calbiochem (San Diego, CA). Celebrex was obtained from Pharmacia Co. (Piscataway, NJ). ON-TARGET smart pool EP1 and EP3 siRNA and ON-TARGET plus siCONTROL Nontargeting pool siRNA were purchased from Dharmacon. The COX-2 IPTG-induced expression plasmid, p-NLR-COX2 was a gift from Dr. W.M. Fu (National Taiwan University) [41]. The IKKα(KM) and IKKβ(KM) mutants were gifts from Dr. H. Nakano (Juntendo University, Tokyo, Japan). The PKCα dominant negative mutant was a gift from Dr. V. Martin (Louis Pasteur de Strasbourg University, France). The c-Src dominant negative mutant was a gift from Dr. S. Parsons (University of Virginia Health System, Charlottesville, VA). The NF-κB luciferase plasmid was purchased from Stratagene (La Jolla, CA) and luciferase assay kit was purchased from Promega (Madison, MA, USA). All other chemicals were obtained from Sigma-Aldrich (St. Louis, MO, USA).

### Cell culture

The human chondrosarcoma cell line (JJ012) was kindly provided by the laboratory of Dr. Sean P Scully (University of Miami School of Medicine, Miami, FL, USA). The JJ012 cells were cultured in DMEM/α-MEM supplemented with 10% fetal bovine serum (FBS) and maintained at 37°C in a humidified atmosphere of 5% CO_2_. The human chondrosarcoma cell line (SW1353) was obtained from the American Type Culture Collection. The cells were cultured in DMEM/α-MEM supplemented with 10% FBS and maintained at 37°C in a humidified atmosphere of 5% CO_2_.

### Migration assay

The migration assay was performed using Transwell (Costar, NY; pore size, 8-μm) in 24-well dishes. Before the migration assay was performed, cells were pretreated for 30 min with different concentrations of inhibitors, including the U73122, GF109203X, PP2, PDTC, TPCK or vehicle control (0.1% DMSO). Approximately 1×10^4 ^cells in 100 μl of serum-free medium were placed in the upper chamber, and 300 μl of the same medium containing PGE_2 _was placed in the lower chamber. The plates were incubated for 24 h at 37°C in 5% CO_2_, then cells were fixed in methanol for 15 min and stained with 0.05% crystal violet in PBS for 15 min. Cells on the upper side of the filters were removed with cotton-tipped swabs, and the filters were washed with PBS. Cells on the underside of the filters were examined and counted under a microscope. Each clone was plated in triplicate in each experiment, and each experiment was repeated at least three times. The number of migrating cells in each experiment was adjusted by the cell viability assay to correct for proliferation effects of the PGE_2 _treatment (corrected migrating cell number = counted migrating cell number/percentage of viable cells) [42].

### Flow Cytometric Analysis

Human chondrosarcoma cells were plated in six-well dishes. The cells were then washed with PBS and detached with trypsin at 37°C. Cells were fixed for 10 min in PBS containing 1% paraformaldehyde. After rinsing in PBS, the cells were incubated with rabbit anti-human antibody against α2, α5, β3, αvβ3, α5β1 or α2β1 integrin (1:100) for 1 h at 4°C. Cells were then washed again and incubated with fluorescein isothiocyanate-conjugated goat anti-rabbit secondary IgG (1:150; Leinco Tec. Inc., St. Louis, MO, USA) for 45 min and analyzed by flow cytometry using FACS Calibur and CellQuest software (BD Biosciences, Palo Alto, CA, USA).

### Western blot analysis

The cellular lysates were prepared as described previously [43]. Proteins were resolved on SDS-PAGE and transferred to Immobilon polyvinyldifluoride (PVDF) membranes. The blots were blocked with 4% BSA for 1 hr at room temperature and then probed with rabbit anti-human antibodies against p-PLCβ3, p-PKCα, p-c-Src or p-p65 (1:1000) for 1 hr at room temperature. After three washes, the blots were subsequently incubated with a donkey anti-rabbit peroxidase-conjugated secondary antibody (1:1000) for 1 hr at room temperature. The blots were visualized by enhanced chemiluminescence using Kodak X-OMAT LS film (Eastman Kodak, Rochester, NY, USA).

### PKC kinase activity assay

PKC activity was assessed by a PKC Kinase Activity Assay Kit according to manufacturer's instructions (Assay Designs, MI). The PKC activity kit is based on a solid-phase ELISA that uses a specific synthetic peptide as a substrate for PKC and a polyclonal antibody that recognized the phosphorylated form of the substrate.

### Reporter assay

The chondrosarcoma cells were transfected with reporter plasmid using Lipofectamine 2000 (Invitrogen) according to the manufacturer's recommendations. Twenty-four hours after transfection, the cells were treated with inhibitors for 30 min, and then PGE_2 _or vehicle was added for another 24 hr. Cell extracts were then prepared, and luciferase and β-galactosidase activities were measured [42,44].

### Quantitative real time PCR

Total RNA was extracted from cancer cells using a TRIzol kit (MDBio, Taipei, Taiwan). The reverse transcription reaction was performed using 2 μg of total RNA that was reversely transcribed into complementary DNA using oligo(dT) primer. The quantitative real time PCR (qPCR) analysis was carried out using a Taqman^® ^one-step PCR Master Mix (Applied Biosystems, CA, USA). One hundred ng of total cDNA were added per 25-μl reaction with sequence-specific primers and Taqman^® ^probes. Sequences for all target gene primers and probes were purchased commercially [GAPDH was used as internal control (Applied Biosystems, CA, USA)]. qPCR assays were carried out in triplicate with an StepOnePlus sequence detection system. The cycling conditions were 10-min polymerase activation at 95°C followed by 40 cycles at 95 °C for 15 s and 60 °C for 60 s. The threshold was set above the non-template control background and within the linear phase of target gene amplification in order to calculate the cycle number at which the transcript was detected (denoted C_T_).

### Patients and specimen preparation

Upon approval by the local ethics committee, specimens of tumor tissue or normal cartilage tissue were obtained from patients, who had been pathologically diagnosed with chondrosarcoma or knee osteoarthritis (the articular cartilage was collected) and had undergone surgical resection at the China Medical University Hospital. The chondrosarcoma patient group consisted of 6 females and 11 males and ranged in age from 22 to 68 years (mean, 48 years). The histologic grade of chondrosarcoma was checked. Cytologically, increased cellularity and cytological atypia are the most important features, and these characteristics are used to determine the grade of the chondrosarcoma [45]. Eleven patients were alive without disease, one was alive with disease, two died without disease, and three died of causes secondary to progressive disease. The overall survival estimate at 5 years was 90%. The recurrence rate for patients with adequate surgical margins was 10%, compared with 75% for patients with inadequate margins. Tissue specimens were ground and then sonicated in a TRIzol kit. The mRNA level was analyzed using qPCR analysis.

For primary cultures from human chondrosarcoma cells, the samples were cleaned of surrounding fat, connective tissue and blood vessels. Thereafter, tissue samples were minced into pieces using a razor blade. Minced samples were transferred into 50 ml Falcon tubes, spun down at 1000 r.p.m. for 5 min and rinsed twice with fresh PBS. Digestion was performed with 1 mg collagenase II/ml PBS at 37°C for 50 min in a shaking water bath. Cell suspension was pipetted up and down at least twice during incubation. After digestion, pure FBS was added to a minimum concentration of 10% to inactivate the collagenase, followed by a centrifugation step as described above. Cells were then cultured in Dulbecco's modified Eagle's medium/α-modified Eagle medium supplemented with 10% FBS and maintained at 37°C in a humidified atmosphere of 5% CO_2 _[46].

Primary cultures of human chondrocytes were isolated from articular cartilage as described previously [47]. The cells were grown in plastic cell culture dishes in 95% air-5% CO2 with Dulbecco's modified Eagle's medium which was supplemented with 10% FBS, 2 mM-glutamine, penicillin (100 U/ml) and streptomycin (100 μg/ml).

### Statistics

The values given are means ± S.E.M. The significance of difference between the experimental groups and controls was assessed by Student's t test. The difference was considered significant if the *p *value was < 0.05.

## Competing interests

The authors declare that they have no competing interests.

## Authors' contributions

Conceived and designed the experiments: CT

Performed the experiments: JL, YF and CC

Analyzed the data and provided the suggests: CHu, HC, WY, CHs and LJ.

All authors read and approved the final manuscript.

## Supplementary Material

Additional file 1 **PLC/PKC/c-Src and NF-κB pathways are involved in PGE_2_-mediated cell migration in human chondrosarcoma cells**. (A) SW1353 cells were incubated with various concentrations of PGE_2_, and *in vitro *migration activity measured with the Transwell after 24 hr showed all supported the cell migration in a dose-dependent way. SW1353 cells were pretreated for 30 min with U73122, GF109203X, PP2 (B), PDTC, TPCK (C) and α2β1 integrin mAb (E) followed by stimulation with PGE_2_, and *in vitro *migration was measured with the Transwell after 24 hr. SW1353 cells were transfection for 24 hr with IKKα, IKKβ, PKC and c-Src mutant or PLC siRNA followed by stimulation with PGE_2_, and *in vitro *migration was measured with the Transwell after 24 hr. Results are expressed as the mean ± S.E. *, p < 0.05 compared with control; #, p < 0.05 compared with PGE_2_-treated group Click here for file

Additional file 2 **PLC/PKC/c-Src and NF-κB pathways are involved in PGE_2_-mediated integrin up-regulation in human chondrosarcoma cells**. (A) SW1353 cells were incubated with PGE_2 _for 24 hr, and the cells surface α5, β3, α5β1, αvβ3 and α2β1 integrin was determined using flow cytometry. (B) SW1353 cells were pretreated for 30 min with U73122, GF109203X, PP2, PDTC and TPCK followed by stimulation with PGE_2_, and cells surface α2β1 integrin was determined using flow cytometry. Results are expressed as the mean ± S.E. *, p < 0.05 compared with control; #, p < 0.05 compared with PGE_2_-treated group Click here for file

Additional file 3 **EP1/PLC/PKC/c-Src and NF-κB pathways are involved in PGE_2_-mediated cell invasion in human chondrosarcoma cells**. JJ012 cells were pretreated for 30 min with α2β1 integrin mAb, U73122, GF109203X, PP2, PDTC and TPCK or transfection for 24 hr with EP1 siRNA followed by stimulation with PGE_2_, and *in vitro *invasion was measured with the Transwell [filters were precoated with Matrigel basement membrane matrix (BD Biosciences, Bedford, MA)) after 24 hr. Results are expressed as the mean ± S.E. *, p < 0.05 compared with control; #, p < 0.05 compared with PGE_2_-treated group Click here for file
