# Supplementary figures and images for: Cyclooxygenase-2 enhances α2β1 integrin expression and cell migration via EP1 dependent signaling pathway in human chondrosarcoma cells
Source: Mol Cancer. 2010 Feb 23;9:43. doi: 10.1186/1476-4598-9-43 (PMC2837621; doi:10.1186/1476-4598-9-43)

**
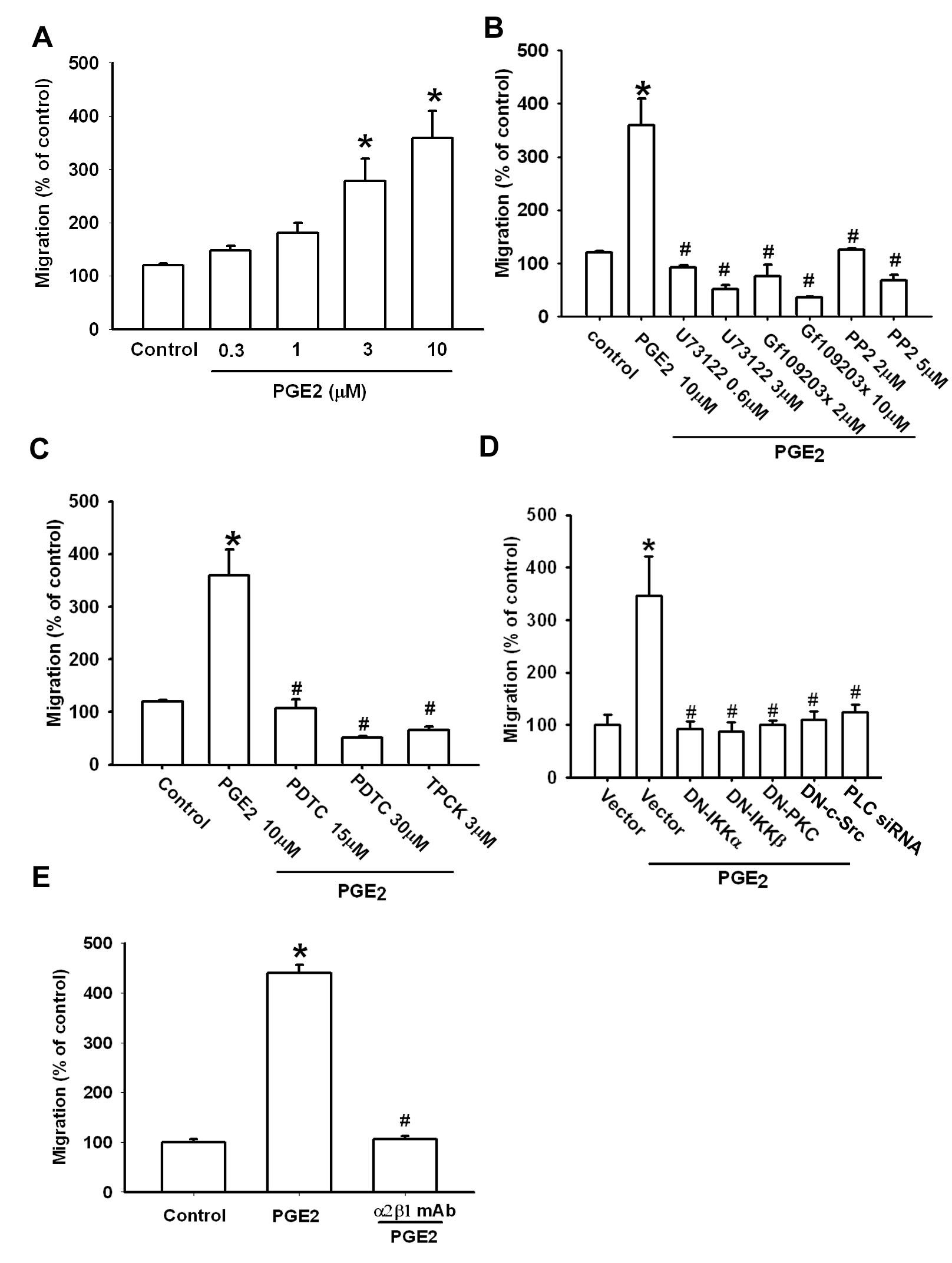
**

Supplement: Additional file 1 — PLC/PKC/c-Src and NF-κB pathways are involved in PGE2-mediated cell migration in human chondrosarcoma cells. (A) SW1353 cells were incubated with various concentrations of PGE2, and in vitro migration activity measured with the Transwell after 24 hr showed all supported the cell migration in a dose-dependent way. SW1353 cells were pretreated for 30 min with U73122, GF109203X, PP2 (B), PDTC, TPCK (C) and α2β1 integrin mAb (E) followed by stimulation with PGE2, and in vitro migration was measured with the Transwell after 24 hr. SW1353 cells were transfection for 24 hr with IKKα, IKKβ, PKC and c-Src mutant or PLC siRNA followed by stimulation with PGE2, and in vitro migration was measured with the Transwell after 24 hr. Results are expressed as the mean ± S.E. *, p < 0.05 compared with control; #, p < 0.05 compared with PGE2-treated group [file 1476-4598-9-43-S1.DOC]

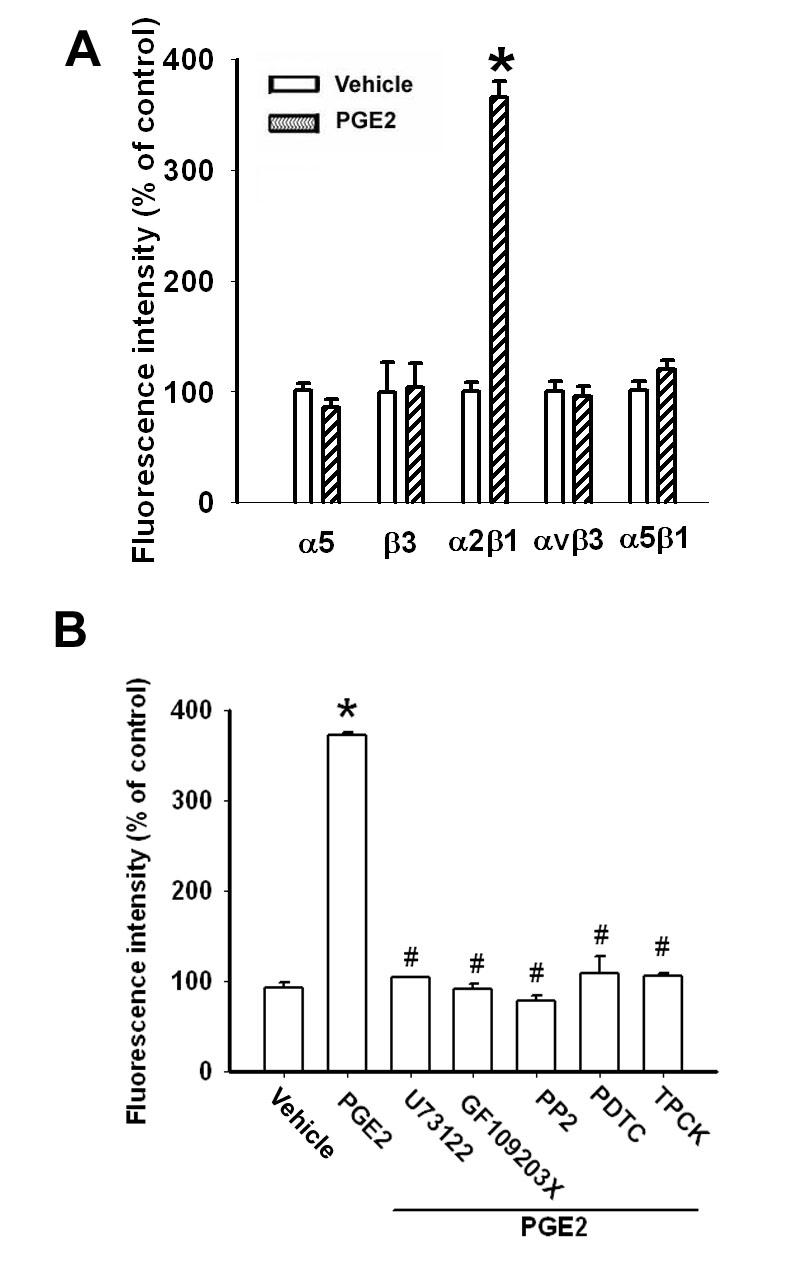

Supplement: Additional file 2 — PLC/PKC/c-Src and NF-κB pathways are involved in PGE2-mediated integrin up-regulation in human chondrosarcoma cells. (A) SW1353 cells were incubated with PGE2 for 24 hr, and the cells surface α5, β3, α5β1, αvβ3 and α2β1 integrin was determined using flow cytometry. (B) SW1353 cells were pretreated for 30 min with U73122, GF109203X, PP2, PDTC and TPCK followed by stimulation with PGE2, and cells surface α2β1 integrin was determined using flow cytometry. Results are expressed as the mean ± S.E. *, p < 0.05 compared with control; #, p < 0.05 compared with PGE2-treated group [file 1476-4598-9-43-S2.DOC]

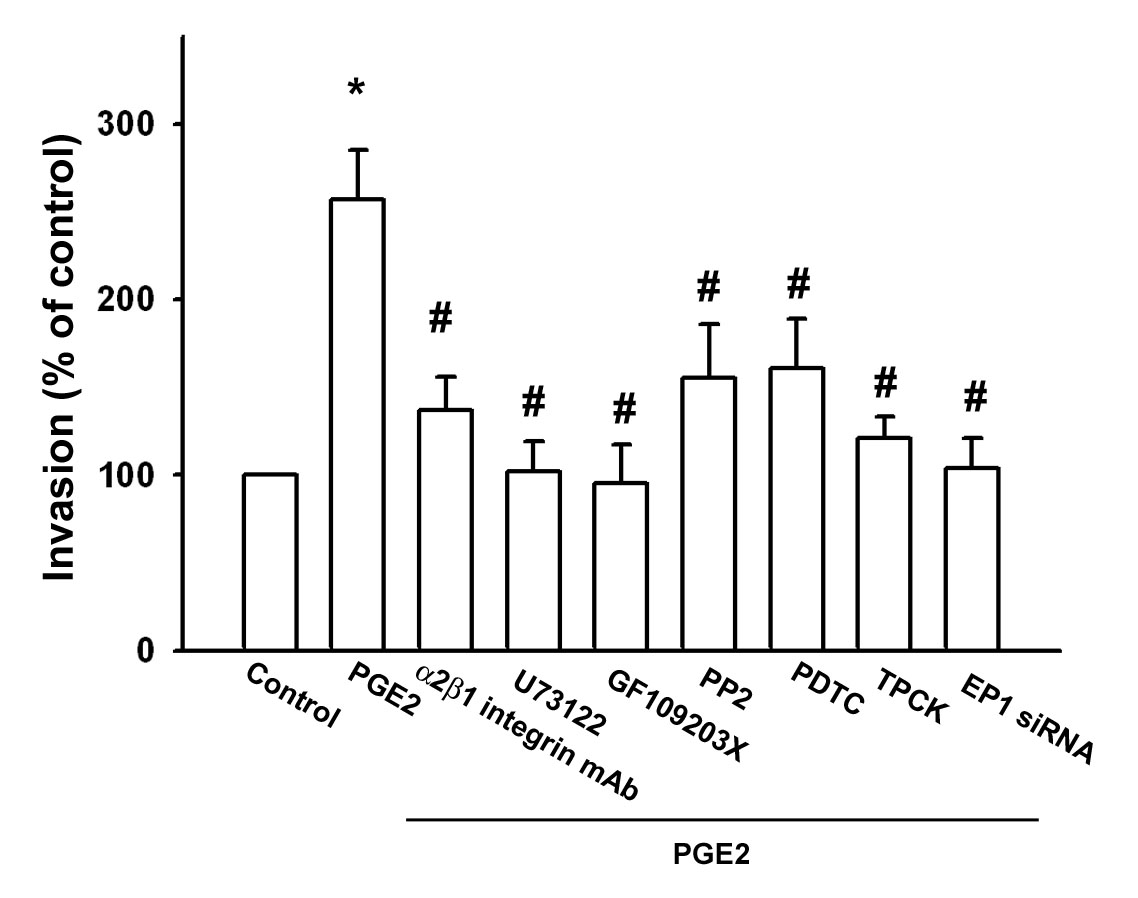

Supplement: Additional file 3 — EP1/PLC/PKC/c-Src and NF-κB pathways are involved in PGE2-mediated cell invasion in human chondrosarcoma cells. JJ012 cells were pretreated for 30 min with α2β1 integrin mAb, U73122, GF109203X, PP2, PDTC and TPCK or transfection for 24 hr with EP1 siRNA followed by stimulation with PGE2, and in vitro invasion was measured with the Transwell [filters were precoated with Matrigel basement membrane matrix (BD Biosciences, Bedford, MA)) after 24 hr. Results are expressed as the mean ± S.E. *, p < 0.05 compared with control; #, p < 0.05 compared with PGE2-treated group [file 1476-4598-9-43-S3.DOC]
